# Proteome Profiling of *S. cerevisiae* Strains Lacking the Ubiquitin-Conjugating Enzymes Ubc4 and Ubc5 During Exponential Growth and After Heat Shock Treatment

**DOI:** 10.3390/microorganisms12112235

**Published:** 2024-11-05

**Authors:** Valentina Rossio, Xinyue Liu, Joao A. Paulo

**Affiliations:** Department of Cell Biology, Harvard Medical School, Boston, MA 02115, USA; xinyue_liu@hms.harvard.edu

**Keywords:** TMT proteomics, ubiquitin–proteasome system, *S. cerevisiae*, heat shock, ubiquitin-conjugating enzymes

## Abstract

The Ubiquitin–Proteasome System (UPS) governs numerous cellular processes by modulating protein stability and activity via the conjugation of the small protein ubiquitin, either as a single molecule or as linkages with distinct functions. Dysregulation of the UPS has been associated with many diseases, including neurodegenerative and neurodevelopmental diseases, as well as cancer. Ubiquitin-conjugating enzymes (E2s) are important players of the UPS that work together with ubiquitin ligases (E3s) to promote substrate ubiquitylation. In this study, we conduct a comparative proteome-wide abundance profiling of *S. cerevisiae* cells during the exponential growth phase with and without heat shock treatment. We focus on cells with deletions of the two highly homologous E2s, *UBC4* or *UBC5*, and use isobaric tag-based quantitative mass spectrometry to elucidate differences and similarities in their proteomic profiles. Our analysis revealed that the deletion of Ubc4 has a stronger effect on the proteome compared to the deletion of Ubc5, particularly in exponentially growing cells. In contrast, the effect on the proteome of deleting Ubc5 becomes evident only after heat shock, and even then, it remains minor compared to Ubc4. Furthermore, we identified proteins increasing in the absence of each enzyme, which may represent candidate substrates, potentially contributing to a better understanding of their cellular role.

## 1. Introduction

The Ubiquitin–Proteasome System (UPS) controls many cellular functions by attaching ubiquitin to substrates [1,2,3,4,5,6]. A cascade of enzymes composed of the ubiquitin-activating enzyme (E1), the ubiquitin-conjugating enzyme (E2), and the ubiquitin ligase enzyme (E3) is responsible for substrate ubiquitylation [1,2,3,4,5,6]. Ubiquitylation is best known for targeting proteins for proteasomal degradation [4,7]. However, it can also have a non-degradative function and alter the activity or localization of proteins [3,5]. Ubiquitin is attached to substrates as a single molecule or as ubiquitin linkages of different topologies. Ubiquitin linkages consist of ubiquitin molecules linked via one of ubiquitin’s seven acceptor lysine residues (Lys6, Lys11, Lys27, Lys29, Lys33, Lys48, and Lys63) or via the N-terminal amino group of the starting methionine of ubiquitin [3]. Each of these linkages serves distinct cellular functions and contributes to the versatility of the UPS. Lys48 linkages, for example, are considered the canonical signal for proteasomal degradation [8] while Lys63 linkages have been shown to have mostly a non-degradative function such as regulation of vesicular trafficking [9,10,11] and of DNA damage [12].

UPS enzymes of *S. cerevisiae* closely resemble their mammalian counterparts, and the regulation of proteins through ubiquitylation is often evolutionarily conserved [8]. The ubiquitin-conjugating enzymes are central components of the UPS [13,14]. These enzymes work together with ubiquitin ligases to ubiquitylate substrates and are largely responsible for the type of ubiquitin linkages that are selectively attached to substrates. Among them, Ubch5 enzymes are the most extensive subfamily, with multiple members present in many species. Human cells, for instance, have four members (Ube2D1-4 or Ubch5a-d) [15], while yeast cells have only Ubc4 and Ubc5 [16]. The human Ubch5 enzymes are involved in many cellular pathways, such as the control of receptor tyrosine kinase activity, TGFB signaling and DNA modification [15]. Ubch5 enzymes exhibit a high degree of similarity across species. The yeast Ubc4 and Ubc5 possess 80% sequence identity with the human homolog Ubch5 [17].

Ubc4 and Ubc5 have been implicated in the degradation of newly translated, misfolded proteins in *S. cerevisiae* [17,18,19]. While Ubc4 is expressed at much higher levels than Ubc5 in exponentially growing cells, Ubc5 is induced in stationary cells [17]. The high level of Ubc4, compared to Ubc5, could explain growth defects displayed by *ubc4Δ* cells and sensitivity to different stresses, such as heat shock [17] (which is investigated here) or drugs that interfere with translation (e.g., hygromycin B and cycloheximide) [19], while *ubc5Δ* cells do not (Table S1). Furthermore, only Ubc4, and not Ubc5, has been shown to work with many E3s, such as the Anaphase Promoting Complex [20], Rsp5 [21] and the SCF complex [22]. *Ubc4Δ ubc5Δ* double mutant cells grow poorly [17,23] or are inviable in some genetic backgrounds [24]; in fact, these two E2s may be functionally redundant [24].

Our goal is to acquire a deeper insight into the function of these two enzymes and to understand their role in regulating proteome stability. Here, we used isobaric tag-based quantitative mass spectrometry (tandem mass tag or “TMT”) to profile and compare protein abundance on a proteome-wide scale for cells lacking *UBC4* or *UBC5* under standard growth conditions and after heat shock treatment. We observed that the loss of Ubc4 induced more substantial changes at the proteome level, compared to the loss of Ubc5 especially in exponentially growing cells. However, after heat shock treatment, proteome-level changes also become evident in the absence of Ubc5, although the effect remains minor compared to Ubc4. We also discovered that after heat shock treatment, only Ubc4, but not Ubc5, was responsible for the increase in Lys48 and Lys11 ubiquitin linkages, suggesting that Ubc4 has a major role in promoting their assembly compared to Ubc5.

## 2. Material and Methods

### 2.1. Materials

The reagents used in this work are commercially available. The protease inhibitors and the BCA kit used for cell lysate preparation were from ThermoFisher Scientific (Rockford, IL, USA). Trypsin and Lys-C proteases for protein digestion were acquired from ThermoFisher Scientific (Rockford, IL, USA) and Fujifilm Wako (Richmond, VA, USA), respectively. Reagents used for proteomic sample preparation were the following: mass spectrometry-grade water and organic solvents (J.T. Baker; Center Valley, PA, USA), tandem mass tag (TMTpro) isobaric reagents (ThermoFisher Scientific; Rockford, IL, USA); StageTip Empore-C18 disks were obtained from CDSanalytical (Oxford, PA, USA), while Sep-Pak cartridges (50 mg) were purchased from Waters (Milford, MA, USA).

### 2.2. Yeast Strains, Growth Conditions and Protein Extraction

All yeast strains used in this study are isogenic to W303 (*ade2-1, trp1-1, leu2-3, 112, his3-11, 15, ura3*). Standard yeast genetics were used to generate *ubc4Δ* and *ubc5Δ* strains [25]. Triplicate cultures of wild-type, *ubc4Δ* and *ubc5Δ* cells were grown overnight at 25 °C in YEPD medium (1% yeast extract, 2% bactopeptone, 50 mg/L adenine, 2% glucose). The next day, cultures were diluted with fresh medium to OD = 0.3 (wild-type and *ubc5Δ*) or OD = 0.4 (*ubc4Δ* and grown at 25 °C until the mid-exponential phase (OD ~ 1) or grown to OD ~ 0.7 at 25 °C before they were subjected to a severe heat shock treatment (42 °C for 80 min) as in Muhlhofer et al., 2019 [26]. Cells were collected by centrifugation at 2000× *g* for 2 min, rinsed with 1 mL water, flash-frozen in liquid nitrogen and stored at −80 °C until mass spectrometry-based proteome analysis. Cell lysis and protein extraction were performed, as described previously in detail [27]. Briefly, cell pellets were resuspended in lysis buffer (8 M urea, 200 mM EPPS (4-(2-hydroxyethyl)-1-piperazinepropanesulfonic acid), pH 8.5 supplemented with protease inhibitors) and lysed by bead-beating (five cycles of 30 s with beating alternating on and off) in the cold room. Protein concentration was determined using a BCA assay performed according to the manufacturer’s instructions. Proteins were reduced with 5 mM tris (2-carboxyethyl)phosphine (TCEP) for 20 min, alkylated with 10 mM NEM for 20 min (in the dark), and finally quenched with 10 mM dithiothreitol (DTT) for 20 min (in the dark). All reactions were incubated at room temperature. A total of 100 µg of protein from each sample was precipitated by chloroform–methanol precipitation [28].

### 2.3. Protein Digestion, TMT Labeling, and Sample Processing

Samples were digested using Lys-C (overnight at 24 °C) and trypsin (6 h at 37 °C). A total of 1 µg of each enzyme was used per 100 µg of protein. A final volume of 30% acetonitrile was added to each digest followed by the addition of specified tandem mass tag (TMTpro) labeling reagents [29]. A total of 50 µg of peptide for each sample was labeled with 120 µg of the appropriate TMTpro reagent as follows: wt triplicates 25 °C: 126, 127n, and 127c; *ubc4Δ* triplicates 25 °C: 128n, 128c, and 129n; *ubc5Δ* triplicates 25 °C: 129, 130n, and 130c; wt triplicates heat shock: 131, 131c, and 132n; *ubc4Δ* triplicates heat shock: 132, 133n, and 133c; *ubc5Δ* triplicates heat shock: 134n, 134c, and 135c. Samples were incubated for one hour at room temperature. Before continuing sample processing, ~1 µg of peptide was collected from each sample, mixed, and desalted via StageTip to verify labeling efficiency (ensuring that it was >97%) [30,31]. Hydroxylamine (final concentration of ~0.3%) was added to each sample to quench the labeling reaction. Samples were incubated at room temperature for 15 min. Then, samples were pooled 1:1 and desalted using a 50 mg Sep-Pak solid-phase extraction column. Fractionation was performed with a basic pH reversed-phase (BPRP) HPLC. An Agilent 1260 pump (Lexington, MA, USA) with an Agilent 300 Extend C18 column (3.5 μm particles, 2.1 mm ID, and 250 mm in length) was used. Peptides were fractionated by applying a 50 min gradient that is linear from 5% to 35% acetonitrile in 10 mM ammonium bicarbonate pH 8 and at a flow rate of 0.25 mL/min. We collected 96 fractions that we concatenated and consolidated down to 24 superfractions from where we obtained two sets of 12 non-adjacent superfractions [32]. We acidified the superfractions with formic acid to a concentration of 1% followed by vacuum centrifugation. Each superfraction was desalted via StageTip, dried again by vacuum centrifugation, and reconstituted in 5% acetonitrile, 5% formic acid.

### 2.4. Mass Spectrometry Data Acquisition and Processing

Mass spectrometric data were acquired on an Orbitrap Fusion Lumos mass spectrometer, which was in line with a Proxeon NanoLC-1200 UHPLC and a FAIMSpro interface. A 100 μm capillary column was manufactured in-lab and packed with 35 cm of C18 beads (Accucore150, 2.6 μm, 150 Å; ThermoFisher Scientific). Data were collected over a 90 min gradient. The scan sequence began with an MS1 spectrum (Orbitrap analysis, resolution 60,000, 400–1600 Th, automatic gain control (AGC) target is set to “100%”, maximum injection time set to 50 ms). The hrMS2 stage consisted of fragmentation by higher energy collisional dissociation (HCD, normalized collision energy 36%) and analysis using the Orbitrap (AGC 200%, maximum injection time 120 ms, isolation window 0.7 Th, resolution 50,000). Data were acquired using the FAIMSpro interface, where the dispersion voltage (DV) was set to 5000V, and the compensation voltages (CVs) were set at −40 V, −60 V, and −80 V. The TopSpeed parameter was set at 1 s per CV.

Spectra were converted to mzXML via MSconvert [33], after which database searching included all *S. cerevisiae* entries from UniProt (the same database as used for RTS, above) and all protein sequences in that database in the reverse order. Searches were performed using a 50 ppm precursor ion tolerance and a product ion tolerance of 0.03 Da to maximize sensitivity in conjunction with Comet database searching and linear discriminant analysis (LDA) while considering the following parameters: XCorr, ΔCn, missed cleavages, peptide length, charge state, and precursor mass accuracy [34,35]. TMT tags on lysine residues and peptide N-termini (+304.207 Da) and n-ethylmaleimide modification of cysteines (+125.048 Da) were set as static, whereas oxidation of methionine residues (+15.995 Da) was set as a variable modification. Peptide-spectrum matches (PSMs) were adjusted to a 1% false discovery rate (FDR), and filtering thereof was performed using a linear discriminant analysis to further assemble the dataset to achieve a final protein-level FDR of 1% [36]. Once completed, proteins were quantified by summing reporter ion counts across matching PSMs. Reporter ion intensities were adjusted for the isotopic impurities of the TMT reagents as specified by the manufacturer. The signal-to-noise (S/N) measurements of peptides assigned to each protein were summed and normalized such that the sum of the signal for all proteins in each channel was equal, thereby correcting for unequal protein loading (i.e., column normalization). Finally, each protein abundance measurement was represented as a percentage of the total, in that the summed S/N for that protein across all channels was 100, thus providing a relative abundance (RA) measurement. We determined protein abundance alterations to be statistically significant if meeting a fold change cutoff |log_2_ ratio| > 0.5 and *p*-value of less than 0.01.

## 3. Results

### 3.1. Comparative Proteome Abundance Profiling of Yeast Deletion Strains of the Homologous Ubiquitin-Conjugating Enzymes UBC4 and UBC5

**Figure 1:**
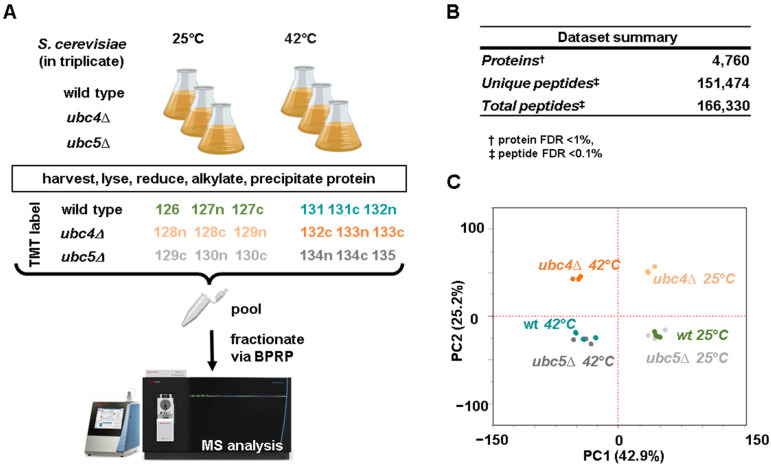
Experimental workflow, summary of the dataset, and principal component analysis. (**A**) Wild-type, *ubc4Δ* and *ubc5Δ S. cerevisiae* cells were grown in triplicate in the exponential phase (24 °C) or subjected to heat shock treatment (42 °C for 80 min). Cells were harvested and processed for mass spectrometry analysis. In brief, yeast cells were lysed, and total protein was extracted and digested. The subsequent peptides were labeled with tandem mass tag (TMTpro) reagents, as indicated, pooled 1:1, and fractionated by basic pH reversed-phase (BPRP) HPLC prior to mass spectrometry analysis. This panel has been assembled, in part, using Biorender.com. (**B**) Summary of the dataset. (**C**) Principal component analysis (PCA) of the dataset highlighting the clustering of the replicates.

We employed isobaric tag-based quantitative proteomics to compare the proteomes of two *S. cerevisiae* strains lacking each of the two highly homologous ubiquitin-conjugating enzymes, *UBC4* and *UBC5,* with wild-type cells, both during exponential cell growth and after heat shock treatment (42 °C for 80 min). Biological triplicate samples were collected, combined in a single TMTpro18-plex experiment and processed using the SL-TMT method [37] (Figure 1A). We quantified 4760 proteins (at a false discovery rate of 1%) across all samples (Figure 1B). These proteins were inferred from 151,474 unique peptides and from a total of 166,330 peptides (Tables S2 and S3). We conducted a principal component analysis (PCA) of the dataset, revealing tight clustering of the replicates under all tested conditions (Figure 1C). PC1 explained more than 42% of the variance, while PC2 explained 25% of the variance. Furthermore, the second principal component highlighted the similarity between the proteome of wild-type and *ubc5Δ* cells compared to that of *ubc4Δ* cells.

**Figure 2:**
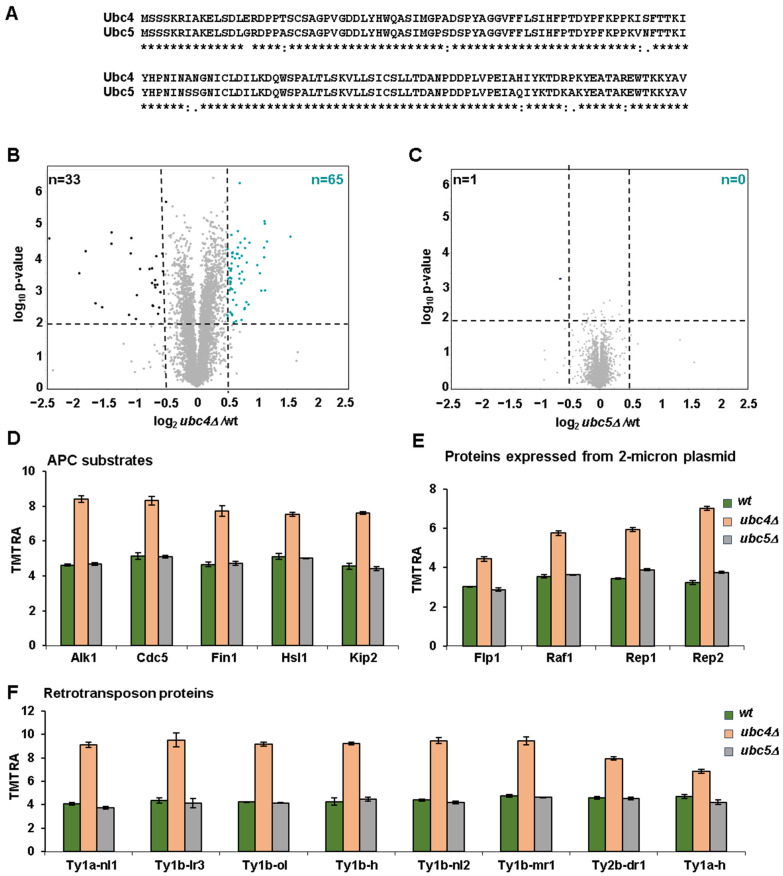
Proteome-wide profiling of differentially abundant proteins in exponentially growing *ubc4Δ* and *ubc5Δ* cells compared to wild-type cells and potential candidate substrates of Ubc4. (**A**) Protein sequence alignment of the two ubiquitin-conjugating enzymes Ubc4 and Ubc5. The volcano plots illustrate differentially abundant proteins (i.e., |log_2_ ratio| > 0.5 and *p*-value < 0.01) between (**B**) wild-type and *ubc4Δ* or (**C**) wild-type and *ubc5Δ* cells. *p*-values are uncorrected two-sided student *t*-tests. Proteins with abundance measurements that are higher in *ubc4Δ* cells are highlighted, such as (**D**) substrates of the Anaphase Promoting Complex (APC), (**E**) proteins expressed from the 2-micron plasmid, and (**F**) retrotransposon proteins. RA: relative abundance. Error bars represent the standard deviation between replicates for panel D through F. TMT RA, tandem mass tag relative abundance.

We choose to compare the proteomes of *UBC4* and *UBC5* knock-out cells, with the hypothesis that proteome differences exist between the two strains despite the fact that these two E2s are nearly identical, having a sequence identity of 92% and differing by only 11 amino acid residues (Figure 2A). The deletion strains were confirmed both by PCR and by mass spectrometry analysis of the unique peptides originating from these two E2s (Figure S1A). Some minimal signal was still observed in the deleted strains (Figure S1A), which is due to interference, a common artifact for isobaric tag-based quantitative proteomics [38]. The relative abundance of peptides unique to Ubc4 was similar in wild-type and *ubc5Δ* cells (Figure S1A). However, the relative abundance of the only peptide unique to Ubc5 was higher in *ubc4Δ* cells compared to wild-type cells (Figure S1A). These data suggest that Ubc5 might be upregulated in *ubc4Δ* cells as a compensatory mechanism for the absence of Ubc4.

By comparing the proteome of exponentially growing *ubc4Δ* and *ubc5Δ* cells with that of wild-type cells, we discovered that the deletion of *UBC4* has a more pronounced effect on proteome stability than the deletion of *UBC5*. These data were consistent with our PCA analysis, which underscored the similarity between *ubc5Δ* and wild-type cells (Figure 1C). Specifically, while only one protein was significantly downregulated (log_2_Fc < −0.5, log_10_ *p*-value < 0.01) in *ubc5Δ* cells (Figure 2C), and none was upregulated, 33 proteins were significantly downregulated in *ubc4Δ* cells, and 65 were significantly upregulated compared to wild-type cells (Figure 2B). These proteins are listed in Table S4.

As Ubc4 and Ubc5 are ubiquitin-conjugating enzymes and, together with ubiquitin ligases, promote substrate ubiquitylation, a possibility exists that the proteins, whose levels increase in *ubc4Δ* cells, are candidate substrates of this enzyme that are targeted for degradation. Therefore, first, we searched for proteins known to be substrates of Ubc4, among the list of proteins increasing in *ubc4Δ* cells compared to wild-type. Consistent with Ubc4 being a known E2 of the mitotic ubiquitin ligase Anaphase Promoting Complex (APC) [20], multiple substrates targeted by the APC were among the proteins increasing in *ubc4Δ* cells (Figure 2C). These substrates included the three mitotic kinases Hsl1 [39], Cdc5 [40] and Alk1 [41], as well as two microtubule-binding proteins Kip2 [42] and Fin1 [43] (Figure 2D). We also observed that among the proteins whose levels increased in *ubc4Δ* cells were all the proteins expressed by the 2 micron plasmid [44], specifically the recombinase Flp1, the recombinase activating factor Raf1 and the two replication proteins Rep1 and Rep2 (Figure 2E). An increase in the abundance of the 2 micron plasmid in *ubc4Δ* cells has been observed previously [45], which may suggest that Ubc4 controls the stability of these proteins that are expressed by this plasmid and is responsible for its maintenance. By conducting an enrichment analysis for the proteins significantly upregulated in *ubc4Δ* cells compared to wild-type cells, we uncovered significant enrichment of retrotransposon proteins (Figure S2B) [46]. Specifically, eight of these proteins were significantly increasing in *ubc4Δ* cells compared to wild-type cells (Figure 2F). These findings suggested that Ubc4 could regulate the stability of the retrotransposon proteins, a class of proteins that has an important impact on genome stability and gene regulation [47].

### 3.2. Proteome-Level Profiling of Heat Shock-Induced Changes in Wild-Type Cells and in Cells Lacking UBC4 or UBC5

**Figure 3:**
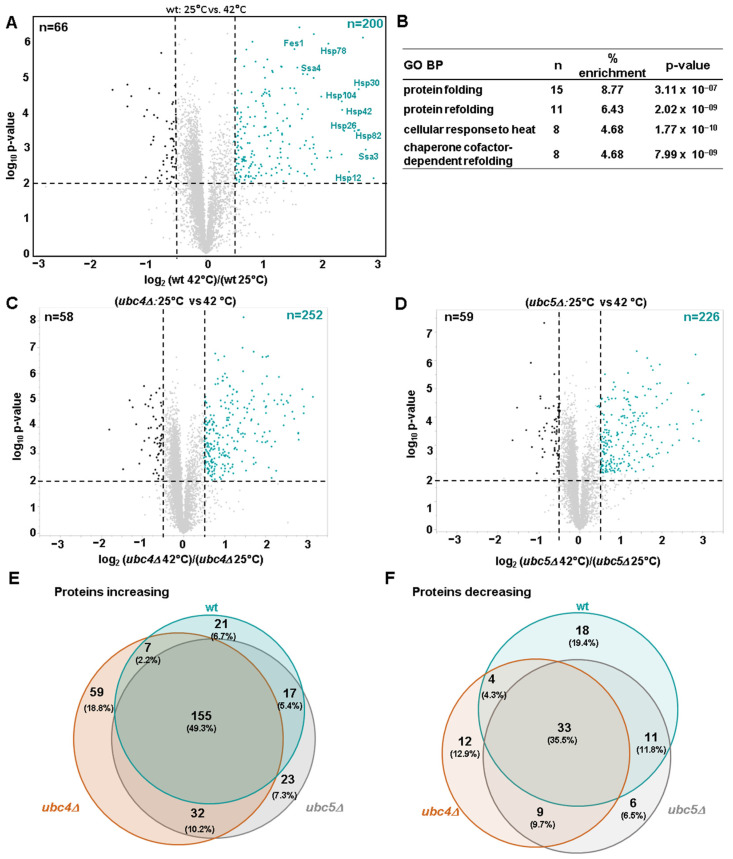
Proteome-wide profiling of differentially abundant proteins in wild-type, *ubc4Δ* and *ubc5Δ* after heat shock treatment. (**A**) The volcano plot illustrates differentially abundant proteins (i.e., |log_2_ ratio| > 0.5, and *p*-value < 0.01) in wild-type cells grown at 25 °C and after heat shock treatment (42 °C for 80 min). (**B**) The top gene ontology (GO) biological processes (BP) terms associated with the proteins increasing after heat shock treatment in wild-type cells. The volcano plots illustrate differentially abundant proteins (i.e., |log_2_ ratio| > 0.5, and *p*-value < 0.01) in (**C**) *ubc4Δ* cells and (**D**) *ubc5Δ* cells at 25 °C, and after heat shock treatment (42 °C for 80 min). The Venn diagrams show the overlap between the proteins (**E**) increasing and (**F**) decreasing in wt, *ubc4Δ* and *ubc5Δ* cells after heat shock. The Venn diagrams have been created using BioVenn [48].

Exponentially growing wild-type, *ubc4Δ*, and *ubc5Δ* cells were subjected to heat shock treatment by shifting them from 25 °C to 42 °C for 80 min. Proteome abundance profiling identified 200 proteins that were significantly increased in wild-type cells exposed to heat shock, and 66 that were significantly decreased (Figure 3A). As anticipated, the proteins that exhibited the most significant increase in abundance were the heat shock proteins (HSPs) belonging to the HSP70 family (Fes1, Ssa3, Ssa4), the HSP90 family (Hsp82), the HSP100 family (Hsp104) and the small heat shock family (Hsp26 and Hsp42). Additionally, we observed an increase in the mitochondrial heat shock protein Hsp78 and Hsp10, as well as in two heat shock plasma membrane proteins (Hsp30 and Hsp12). These differentially abundant proteins are listed in Table S5. Gene ontology analysis of the proteins that significantly increased after heat shock revealed enrichment of proteins involved in protein folding, protein refolding in response to heat and in protein refolding dependent on a cofactor (Figure 3B). The increase in the heat shock proteins and gene ontology analysis both confirm the effectiveness of the heat shock treatment.

Next, we profiled the effect of deleting *UBC4* or *UBC5* on the global proteome of yeast cells exposed to heat shock. The heat shock-associated alterations in the proteome of *ubc4Δ* and *ubc5Δ* cells were similar to those in wild-type cells. Indeed, the correlation between the alterations in protein levels (log_2_ ratio) induced by heat shock in wild-type cells and *ubc4Δ* or *ubc5Δ* cells was high, having R^2^ values of 0.813 and 0.885, respectively (Figure S2). Proteomic abundance profiling identified 252 and 226 proteins that were significantly increased in *ubc4Δ and ubc5Δ* cells, respectively, and 58 and 59 that were significantly decreased (Figure 3C,D) when comparing the proteome of exponentially growing cells with those subjected to heat shock. We also compared the proteins that significantly increased or decreased after heat shock treatment in the three strains (Figure 3E,F). Most of the differentially abundant proteins varied to a degree in all three strains (155 and 33, respectively). However, specific proteins were differentially abundant in only one or two of the three strains. Proteins that increased specifically in *ubc4Δ* (59), in *ubc5Δ* (23), or in both (32) could be candidate substrates of these E2s targeted for degradation under heat shock stress. Following heat shock, the effect on the proteome induced by Ubc4 was again greater compared to the one induced by Ubc5. In fact, while 112 proteins were differentially abundant in *ubc4Δ* cells, only 90 proteins were differentially abundant in *ubc5Δ* cells compared to wild-type cells. The differentially abundant proteins indicated in these Venn diagrams are listed in Table S6.

### 3.3. Profiling of Ubiquitin Linkages Following Heat Shock Treatment in Wild Type, ubc4Δ and ubc5Δ Cells

In our proteome profiling experiment, we conducted a database search for the GG signature peptide (114.029 Da) as a variable modification on lysine (Lys) residues in the proteomes of wild-type, *ubc4Δ* and *ubc5Δ* cells to detect ubiquitin linkages. This strategy was employed previously to identify lysine ubiquitylation sites on proteins [49] when di-Gly proteomic immunoprecipitation workflows were not yet available. We detected the three most abundant ubiquitin linkages in *S. cerevisiae* [50]: Lys48, Lys11 and Lys63. We did not detect the other four lysine ubiquitin linkages (Lys6, Lys,27, Lys29 and Lys33) likely because of their lower abundance in yeast cells [50].

**Figure 4:**
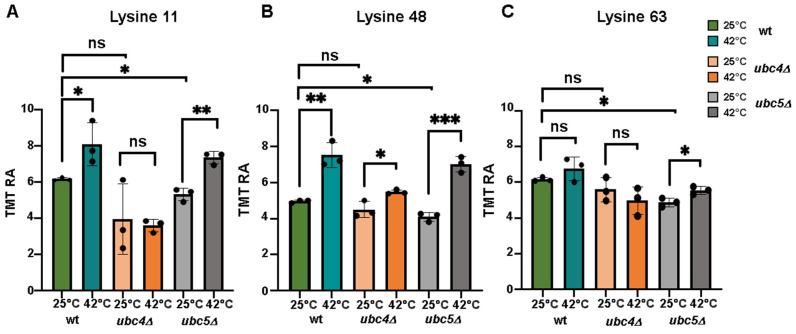
Analysis of the lysine ubiquitin linkages following heat shock. Relative abundance (TMT RA) of the lysine ubiquitin linkages (**A**) Lys11, (**B**) Lys48, and (**C**) Lys43 (detected in the experiment during exponential growth at 25 °C and after heat shock treatment (42 °C) in the strains investigated. Statistical significance was calculated using a two-tailed unpaired *t*-test (ns = *p* > 0.05, * *p*-value < 0.05, ** *p*-value < 0.01, *** *p*-value < 0.001). Dot represents each replicate in that specific condition.

In exponentially growing cells, only the deletion of *UBC5*, but not of *UBC4,* significantly affects the ubiquitin linkages we detected compared to wild-type (Figure 4A–C). We observed a significant increase in both Lys11 and Lys48 ubiquitin linkages after heat shock treatment in wild-type cells (Figure 4A,B), indicating that heat shock induces the formation of these linkages under our experimental conditions. In contrast, Lys63 linkage did not show significant changes (Figure 4C). The increase in Lys48 and Lys11 linkages indicated that more proteins were targeted for proteasomal degradation during heat shock, as these linkages are known to be the canonical proteasome targeting signal [50]. In cells lacking *UBC4*, both Lys11 and Lys48 linkages exhibited a marginal increase compared to wild-type cells, suggesting that Ubc4 was involved in their formation during heat shock treatment. However, the deletion of *UBC5* had no effect on either of these linkages, suggesting that Ubc5 is not involved or has only a minor role in promoting their assembly during heat shock treatment.

## 4. Discussion

Here, we compared proteome abundance profiles between *S. cerevisiae* wild-type cells and cells lacking two highly homologous ubiquitin-conjugating enzymes *UBC4* or *UBC5*. This proteome profiling was conducted both in exponentially growing cells and in cells subjected to heat shock. We observed that in exponentially growing cells, deletion of *UBC4* has a stronger effect on the proteome. In fact, while around 100 proteins were differentially abundant in *ubc4Δ* cells compared to wild-type, only one was differentially abundant in *ubc5Δ* cells.

Our proteome profiling is consistent with the observation that while *UBC4* is expressed in exponentially growing cells, *UBC5* is weakly expressed during exponential growth, with its expression increasing drastically upon transition from the exponential to stationary phase [17]. It is possible that Ubc5 plays a greater role in regulating proteome stability in cells that are in stationary phase, while Ubc4 has a primary role in exponentially growing cells. The role of Ubc5 on the proteome stability of yeast cells growing in stationary phase is a question that we plan to address in future studies. Even if the proteome remained relatively stable in *ubc5Δ* cells, the deletion of *UBC5* significantly decreased the ubiquitin linkages detected in exponentially growing cells compared to wild-type cells. This phenomenon can be due to Ubc5 targeting non-degradative substrates or the fact that the ubiquitin attached by Ubc5 is not sufficient to affect the stability of its substrates.

We postulate that Ubc5 becomes more important only in the absence of Ubc4. Consistent with this, we observed that Ubc5 protein levels increased in *ubc4Δ* cells likely as a compensatory mechanism for the absence of Ubc4, providing evidence of functional redundancy, as has been suggested previously [24]. Another possibility is that Ubc5 activity becomes more prevalent when Ubc4 activity is insufficient to ubiquitylate all proteins that need to be ubiquitylated. In accordance with this hypothesis, after heat shock treatment, we observed that changes in the proteome also become evident in *ubc5Δ* cells, although the effect remains minor compared to cells in which Ubc4 is absent. We also found that only Ubc4, but not Ubc5, was responsible for the increase in Lys48 and Lys11 ubiquitin linkages that were generated during heat shock. The increase in Lys48 and Lys11 linkages is likely due to many proteins being targeted for proteasomal degradation during heat shock, as these linkages are known to be the canonical proteasomal targeting signal [50]. Our analysis suggests that Ubc4 has a major role in assembling these two linkages compared to Ubc5 even if both Ubc4 and Ubc5 have been shown to be important for the degradation of newly translated misfolded proteins [17,18] that originate during heat shock.

One limitation of our study is that the heat shock treatment was conducted by collecting samples at a single time point (after 80 min). It is possible that a longer treatment with heat shock could reveal more changes at the proteome level that we missed in our experimental condition.

Importantly, our proteome analysis confirmed previously identified substrates of Ubc4, including proteins ubiquitylated and targeted for degradation by the Anaphase Promoting Complex, a ubiquitin ligase that partners with Ubc4 [20]. However, many of the proteins that increase in the absence of Ubc4 have not been linked previously with this enzyme. These proteins could be potential candidate substrates of Ubc4, providing insights into its cellular functions. One example is the retrotransposon proteins, which were among those enriched in the absence of Ubc4. These proteins have an important role in regulating gene expression and in ensuring genome stability [51].

Our proteome analysis serves as an important resource for researchers investigating the Ubiquitin–Proteasome System, which regulates many cellular processes. We recommend that subsequent studies include orthogonal and targeted experiments to further validate and complement our study.

Altogether, our study offers the first comparative proteome analysis of *ubc4Δ* and *ubc5Δ* cells under different conditions, highlighting their similarities and differences. Additionally, it identifies candidate substrates for these enzymes, especially for Ubc4, which had a stronger effect on proteome stability.

## Data Availability

RAW files will be made available upon request in addition to the data that have been deposited to the ProteomeXchange Consortium via the PRIDE [34] partner repository with the dataset identifier PXD056103.

## Supplementary Materials

The following supporting information can be downloaded at: https://www.mdpi.com/article/10.3390/microorganisms12112235/s1, Figure S1: Protein abundance profiling of the unique peptides from Ubc4 and Ubc5 in exponentially growing cells and top gene ontology (GO) pathways associated with the proteins that have a higher abundance in *ubc4Δ* cells. Figure S2: Effect of *UBC4* or *UBC5* deletion on the proteome of yeast cells exposed to heat shock compared to wild type yeast cells. Table S1: Characteristics of Ubc4 and Ubc5 in *S. cerevisiae.* Table S2: Proteins quantified in the experiment. Columns include: Protein ID, Gene symbol, Description, Number of Peptides assigned to a given protein, and 18 columns of TMT signal-to-noise values that have been scaled to 100 across all channels. Table S3: Peptides quantified in the experiment. Columns include: Uniprot protein ID, gene symbol, protein description, protein group ID, redundancy, whether peptide is a unique or razor peptide, peptide sequence, and TMT signal-to-noise for all channels. Table S4: Differentially abundant proteins in exponentially growing *ubc4Δ* and *ubc5Δ* cells versus wild type cells. Tabs include data for 1) *ubc4Δ* vs wt and 2) *ubc5Δ* vs wt. Columns include: UniProt Protein ID, gene symbol, protein description, number of peptides per protein, log_2_ fold change, and log_10_ *p*-value (two-sided *t*-test). This table is related to Figure 2B and C. Table S5: Differentially abundant proteins between exponential growth and heat shock in wt, as well as *ubc4Δ* and *ubc5Δ* cells. Tabs include data for (1) wt, (2) *ubc4Δ,* and (3) *ubc5Δ* strains. Columns include: UniProt Protein ID, gene symbol, protein description, number of peptides per protein, log_2_ fold change, and log_10_ *p*-value (two-sided *t*-test). This table is related to Figure 3A–C. Table S6: Overlapping and non-overlapping differentially abundant proteins with respect to heat shock treatment. Tabs include data for proteins with abundance (1) increasing and (2) decreasing with respect to heat shock treatment. Columns list proteins in a given overlap category. This table is related to Figure 3E.
