# Supplementary material for: Proteome Profiling of S. cerevisiae Strains Lacking the Ubiquitin-Conjugating Enzymes Ubc4 and Ubc5 During Exponential Growth and After Heat Shock Treatment
Source: Microorganisms. 2024 Nov 5;12(11):2235. doi: 10.3390/microorganisms12112235 (PMC11596627; doi:10.3390/microorganisms12112235)
Supplement: Supplementary file 1 [file microorganisms-12-02235-s001.zip › Table S1_JP_micro_JP.pdf]

Table S1: List of the properties of Ubc4 and Ubc5 and the physiological consequences of their deletion

	**Ubc4**	**Ubc5**
**Properties**	***-Expression level***<br>High in exponential phase and weak in stationary phase[a]; induced by heat shock[a];<br><br>-***Associated ubiquitin ligases*** Anaphase Promoting Complex[b],<br>Rsp5[c], SCF complex[d], Not4[e];<br><br>***- Interacts with the proteasome*** [f]	***-Expression level***<br>Weak in exponential phase and high in stationary phase[a]; induced by heat shock[a];<br><br>**-Associated ubiquitin ligases**<br>Not4[e];<br><br>***-Interacts with the proteasome***[f]
**Physiological consequences of their deletion**	-Mild growth defect[f];<br><br>-Not temperature sensitive[a];<br><br>-Sensitive to translation inhibitors [f];<br><br>-Sensitive to arsenic treatment[g];	-No growth defect[f] ;<br><br>-Not temperature sensitive[a,f];<br><br>-Insensitive to translation inhibitors[f]<br><br>-Insensitive to arsenic treatment[g];

a Seufert, W.; Jentsch, S. Ubiquitin-Conjugating Enzymes UBC4 and UBC5 Mediate Selective Degradation of Short-Lived and Abnormal Proteins. *The EMBO Journal* 1990, *9* (2), 543–550. https://doi.org/10.1002/j.1460-2075.1990.tb08141;

b Girard, J. R.; Tenthorey, J. L.; Morgan, D. O. An E2 Accessory Domain Increases Affinity for the Anaphase-Promoting Complex and Ensures E2 Competition. *Journal of Biological Chemistry* 2015, *290* (40), 24614–24625. https://doi.org/10.1074/jbc.M115.678193;

c Gwizdek, C.; Hobeika, M.; Kus, B.; Ossareh-Nazari, B.; Dargemont, C.; Rodriguez, M. S. The mRNA Nuclear Export Factor Hpr1 Is Regulated by Rsp5-Mediated Ubiquitylation. *J Biol Chem* 2005, *280* (14), 13401–13405. https://doi.org/10.1074/jbc.C500040200;

d Kus, B. M.; Caldon, C. E.; Andorn-Broza, R.; Edwards, A. M. Functional Interaction of 13 Yeast SCF Complexes with a Set of Yeast E2 Enzymes in Vitro. *Proteins* 2004, *54* (3), 455–467. https://doi.org/10.1002/prot.10620;

e Mulder K., Inagaki A., Cameroni E., Mousson F., Winkler S., De Virgilio C., Collart M, Timmers M. Modulation of Ubc4p/Ubc5p-mediated stress responses by the RING-finger-dependent ubiquitin-protein ligase Not4p in Saccharomyces cerevisiae Genetics 2007 May;176(1):181-92. doi: 10.1534/genetics.106.060640;

f Chuang, S.-M.; Madura, K. Saccharomyces Cerevisiae Ub-Conjugating Enzyme Ubc4 Binds the Proteasome in the Presence of Translationally Damaged Proteins. *Genetics* 2005, *171* (4), 1477–1484. https://doi.org/10.1534/genetics.105.046888;

g Jochem M., Ende L, Isasa M., Ang J., Schnell H., Guerra-Moreno A. , Micoogullari Y., Bhanu M., Gygi SP, Hanna J Targeted Degradation of Glucose Transporters Protects against Arsenic Toxicity Mol Cell Biol 2019 Apr 30;39(10):e00559-18. doi: 10.1128/MCB.00559-18;
